# Relationship Between Misophonia and Obsessive-Compulsive Disorder: Systematic Review and Meta-Analysis

**DOI:** 10.1192/j.eurpsy.2026.11075

**Published:** 2026-07-31

**Authors:** C. Spallarossa, C. Figini, P. Motaghilotf, P. Politi, L. Fusar Poli

**Affiliations:** Department of Brain and Behavioral Sciences, University of Pavia, Pavia, Italy

## Abstract

**Introduction:**

Misophonia is a condition characterized by disproportionate emotional, behavioral, and physiological reactions to specific auditory and/or visual stimuli perceived as harmless by most people. Although not yet included in international diagnostic systems, misophonia has gained growing clinical and scientific interest. Its nosological ambiguity has hindered the understanding of associated psychiatric comorbidities. Among these, obsessive-compulsive disorder (OCD) is most frequently reported. The two conditions share features such as intrusive thoughts, heightened attention to specific stimuli, and avoidance behaviors. However, most individuals with misophonia do not meet full diagnostic criteria for OCD, suggesting that misophonia may represent a distinct yet partially overlapping psychopathological entity. This study systematically reviews and quantitatively analyzes the relationship between misophonia and OCD.

**Objectives:**

To examine the co-occurrence and association between misophonia and OCD through a systematic review and meta-analysis, estimating both the pooled prevalence of OCD in misophonia and the correlation between the two conditions.

**Methods:**

A systematic search of *Web of Science* and *PsycINFO* (January 2025) identified studies including individuals with misophonia and a comorbid psychiatric disorder diagnosed via clinical interview or validated scales. Of 464 screened records, 17 met inclusion criteria (total N = 1 193) (Image 1). Two random-effects meta-analyses were conducted: a **prevalence analysis** (k = 12) and a **correlational analysis** (k = 6). Heterogeneity (I^2^) and publication bias were assessed following PRISMA guidelines.

**Results:**

The **prevalence meta-analysis** (k = 12) showed an aggregated OCD prevalence of **9.2 %** (95 % CI 4.7–17.4) in misophonia samples, with substantial heterogeneity. The **correlational meta-analysis** (k = 6) revealed a **moderate positive correlation** (μ = 0.389, 95 % CI 0.290–0.487, *p* < .001; I^2^ = 64.9 %), consistent with a reliable association across studies. The 95 % prediction interval (0.184–0.594) indicates that this link is expected in future research. No publication bias was detected, supporting the robustness of results (Images 2-3).

**Image 1:**
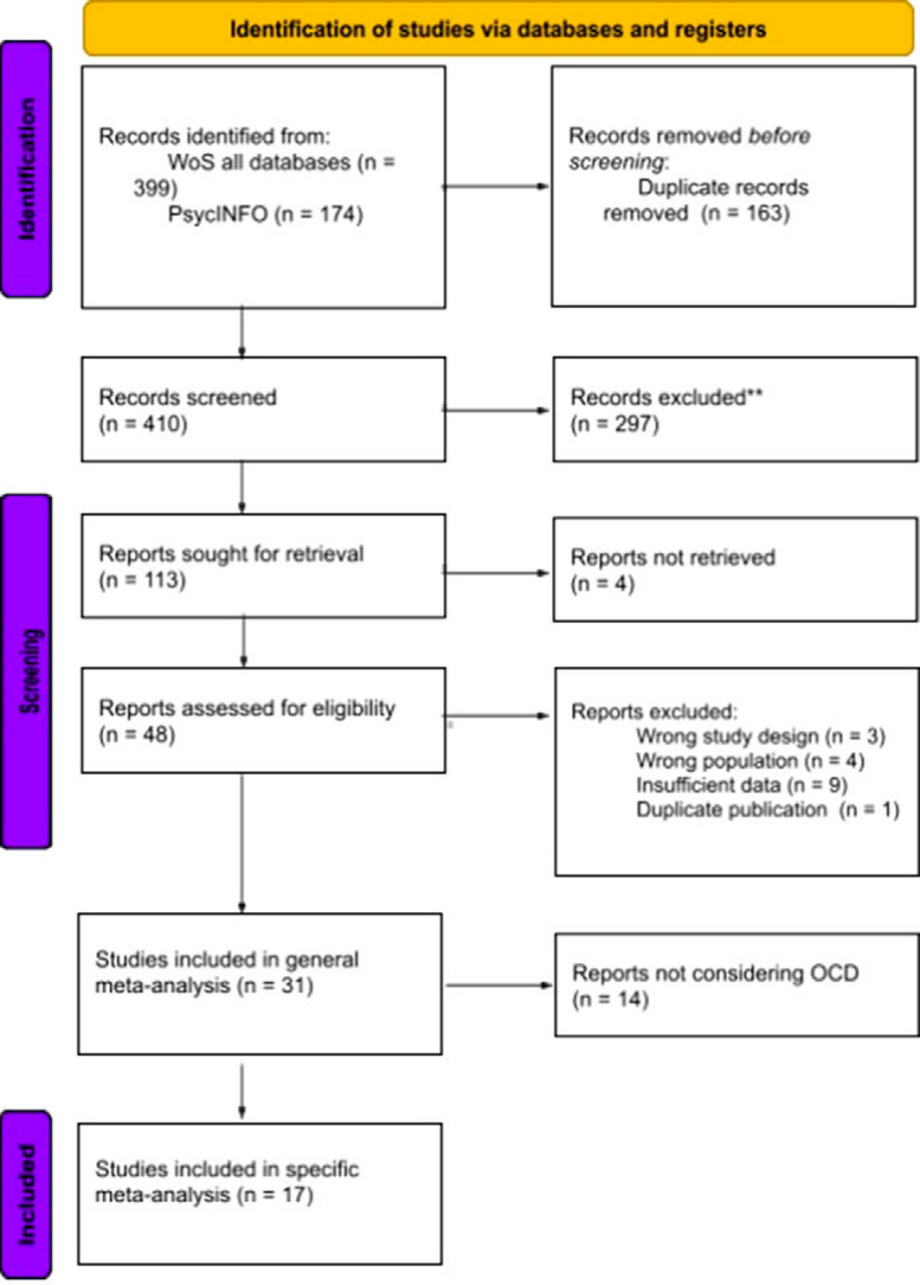
Image 1: Long description.

**Image 2:**
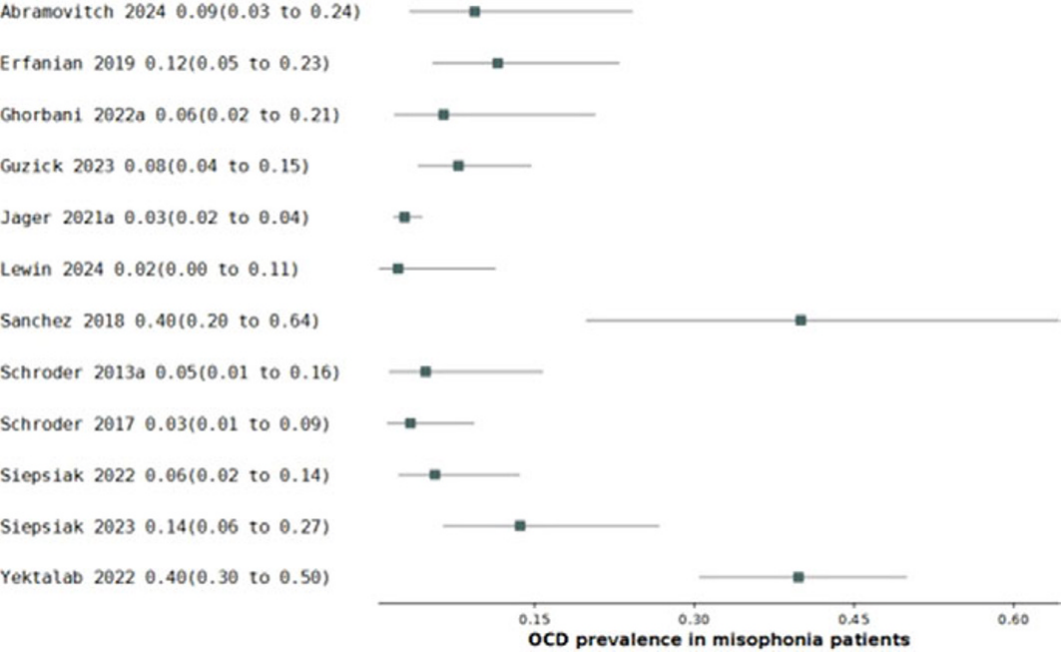
Image 2: Long description.

**Image 3:**
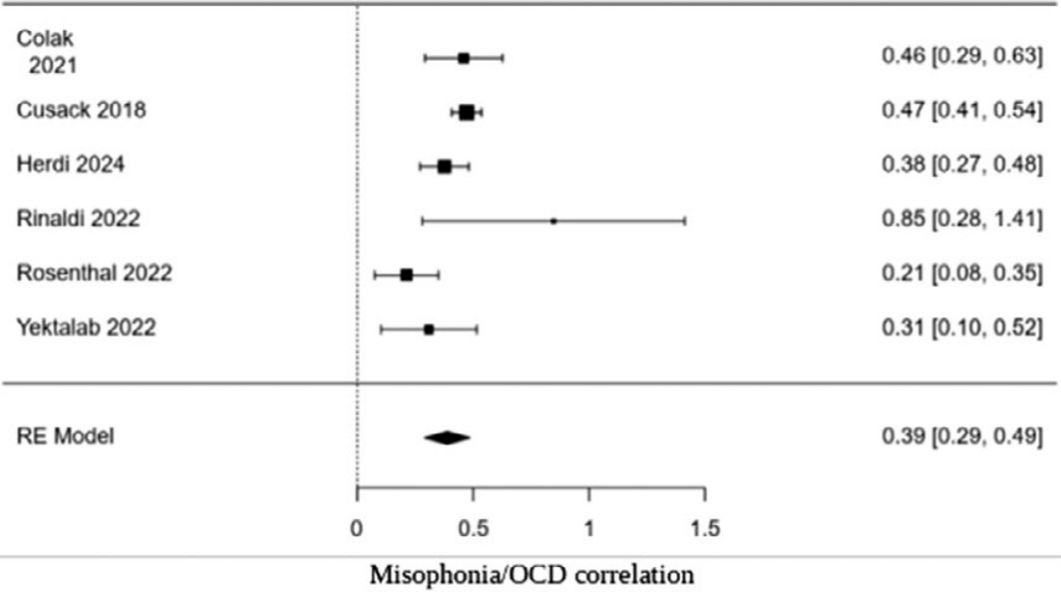
Image 3: Long description.

**Conclusions:**

Current evidence supports a significant, though variable, association between misophonia and OCD, with an average correlation of moderate strength. These results reinforce the hypothesis of **shared psychopathological mechanisms**, possibly involving cognitive-perceptual rigidity and emotional hyper-reactivity. Clinically, obsessive-compulsive symptoms may worsen misophonia and its impact on daily life. Nevertheless, **misophonia presents a unique symptom profile, suggesting it constitutes an independent though related disorder**. Further studies with standardized diagnostic criteria and larger samples are needed to refine its classification and guide targeted interventions.

**Disclosure of Interest:**

None Declared

## Long descriptions

### Image 1: Long description

A vertical flowchart divided into three phases: Identification, Screening, and Included.

1. Identification Phase:

- Top box: Identification of studies via databases and registers.

- Left box: Records identified from W o S all databases (n = 399) and Psyc I N F O (n = 174).

- Right box: Records removed before screening: Duplicate records removed (n = 163).

2. Screening Phase:

- Central box: Records screened (n = 410). An arrow points right to: Records excluded (n = 297).

- Central box: Reports sought for retrieval (n = 113). An arrow points right to: Reports not retrieved (n = 4).

- Central box: Reports assessed for eligibility (n = 48). An arrow points right to: Reports excluded due to Wrong study design (n = 3), Wrong population (n = 4), Insufficient data (n = 9), and Duplicate publication (n = 1).

- Central box: Studies included in general meta-analysis (n = 31). An arrow points right to: Reports not considering O C D (n = 14).

3. Included Phase:

- Final box: Studies included in specific meta-analysis (n = 17).

Navigate back to Image 1:.

### Image 2: Long description

The x-axis is labeled O C D prevalence in misophonia patients with numerical increments at 0.15, 0.30, 0.45, and 0.60. The plot lists twelve studies vertically from top to bottom.

* Abramovitch 2024: 0.09 (0.03 to 0.24). The point is positioned below 0.15 with wide error bars.

* Erfanian 2019: 0.12 (0.05 to 0.23). The point is slightly right of the previous study.

* Ghorbani 2022a: 0.06 (0.02 to 0.21). The point is positioned to the left of 0.15.

* Guzick 2023: 0.08 (0.04 to 0.15). The point is positioned to the left of 0.15.

* Jager 2021a: 0.03 (0.02 to 0.04). A very tight cluster near the zero baseline.

* Lewin 2024: 0.02 (0.00 to 0.11). The point is at the far left edge.

* Sanchez 2018: 0.40 (0.20 to 0.64). A significant outlier with a point at 0.40 and very wide error bars extending past 0.60.

* Schroder 2013a: 0.05 (0.01 to 0.16). The point is near the left baseline.

* Schroder 2017: 0.03 (0.01 to 0.09). The point is near the left baseline.

* Siepsiak 2022: 0.06 (0.02 to 0.14). The point is positioned to the left of 0.15.

* Siepsiak 2023: 0.14 (0.06 to 0.27). The point is just before the 0.15 mark.

* Yektalab 2022: 0.40 (0.30 to 0.50). The point is at 0.40 with moderate error bars.

Navigate back to Image 2:.

### Image 3: Long description

The forest plot displays the Misophonia forward slash O C D correlation on an x-axis ranging from 0 to 1.5. A vertical dashed line indicates the zero baseline.

Individual studies from top to bottom include:

* Colak 2021: Data point at 0.46 with a confidence interval of [0.29, 0.63].

* Cusack 2018: Data point at 0.47 with a confidence interval of [0.41, 0.54].

* Herdi 2024: Data point at 0.38 with a confidence interval of [0.27, 0.48].

* Rinaldi 2022: Data point at 0.85 with a wide confidence interval of [0.28, 1.41].

* Rosenthal 2022: Data point at 0.21 with a confidence interval of [0.08, 0.35].

* Yektalab 2022: Data point at 0.31 with a confidence interval of [0.10, 0.52].

Below a horizontal separator line is the summary statistic:

* R E Model: Represented by a diamond shape centered at 0.39 with a confidence interval of [0.29, 0.49].

Navigate back to Image 3:.

